# Randomized Controlled Trial of Darbepoetin α Versus Continuous Erythropoietin Receptor Activator Injected Subcutaneously Once Every Four Weeks in Patients with Chronic Kidney Disease at the Pre-Dialysis Stage

**DOI:** 10.3390/ijms161226229

**Published:** 2015-12-18

**Authors:** Tetsuya Furukawa, Kazuyoshi Okada, Masanori Abe, Ritsukou Tei, Osamu Oikawa, Noriaki Maruyama, Takashi Maruyama

**Affiliations:** Division of Nephrology, Hypertension and Endocrinology, Department of Medicine, Nihon University School of Medicine, 30-1 Oyaguchi-kamichou, Itabashi-ku, Tokyo 173-8610, Japan; tetsuf0605@gmail.com (T.F.); abe.masanori@nihon-u.ac.jp (M.A.); haru_li_huang@yahoo.co.jp (R.T.); oikawa.osamu@nihon-u.ac.jp (O.O.); maruyama.noriaki@nihon-u.ac.jp (N.M.); maruyama.takashi@nihon-u.ac.jp (T.M.)

**Keywords:** chronic kidney disease, erythropoiesis-stimulating agent, darbepoetin α, continuous erythropoietin receptor activator, renal anemia

## Abstract

Continuous erythropoietin receptor activator (CERA) seems to maintain a stable hemoglobin (Hb) level because its half-life is longer than darbepoetin α (DA). Twenty chronic kidney disease (CKD) patients at the pre-dialysis stage who had been administered DA for over 24 weeks were randomly assigned to receive subcutaneous CERA or DA once every four weeks during 48 weeks. In both groups, the rate of achievement of target Hb level changed from 70% to 100% in weeks 0 to 48, with no significant difference between the groups. Compared with week 0, the Hb level was significantly increased from week 24 in the DA group and from week 8 in the CERA group. In addition, the reticulocyte count was significantly increased from week 4 in the CERA group compared with the DA group. There was no significant difference in the levels of estimated glomerular filtration rate and iron status between both groups. Because of the small number of patients in this study, only limited conclusions can be drawn. However, the results suggest that subcutaneous administration of DA or CERA once every four weeks to predialysis patients has similar effects on achievement of target Hb levels.

## 1. Introduction

The number of chronic kidney disease (CKD) patients who progress to end-stage renal disease (ESRD) has increased recently. In addition to potential progression to ESRD, CKD is also a risk factor for cardiovascular disease and death. It is therefore a growing problem worldwide [1,2,3,4]. Current medications can effectively treat CKD, especially when intervention is started early. Anemia progresses along with deterioration of renal function and is associated with significant morbidity and mortality. The depression of serum erythropoietin level is inappropriate given the severity of anemia in CKD patients. It was anticipated that pharmacological replacement with human recombinant erythropoietin (r-HuEPO) would stimulate erythropoiesis and correct the anemia [5]. Although there is considerable theoretical concern that increasing hemoglobin (Hb) level could accelerate the progression of renal dysfunction, several studies have demonstrated the safety and efficacy of r-HuEPO in the correction of anemia in predialysis patients, without causing a decline in renal function [6,7,8].

Despite the proven efficacy of r-HuEPO, advancement in the treatment of anemia through the development of longer-acting erythropoietin analogues has been expected. The first to be synthesized was darbepoetin α (DA), a second-generation erythropoiesis-stimulating agent (ESA). The molecular weight of darbepoetin α is 37.1 kDa, compared with 30.4 kDa for r-HuEPO, and its elimination half-life in man after an intravenous injection is about three-fold longer (25.3 *vs.* 8.5 h for epoetin α). More recently, a third-generation ESA, continuous erythropoietin receptor activator (CERA) was created. The molecular weight of CERA is about 60 kDa and its elimination half-life in man was considerably increased to about 130 h intravenously. In addition, the subcutaneous elimination half-life is about 48.8 h for DA and about 130 h for CERA, suggesting that CERA is more suitable for patients with CKD, particularly those who do not yet need dialysis [5,9].

MacDougall *et al.* reported a study in which ESA-naïve CKD patients not on dialysis were assigned to receive subcutaneous CERA (0.6 μg/kg every two weeks) or subcutaneous DA (0.45 μg/kg per week). This study consisted of an 18-week correction period for dosage titration and Hb correction, followed by a 10-week evaluation period for efficacy assessments. In addition, CERA and DA responders were allowed to continue on the same drug during the additional 24-week extension period. CERA once every two weeks was as effective as DA once weekly for correcting anemia in the evaluation period [10]. Roger *et al.* reported that CERA (1.2 μg/kg once every four weeks) successfully corrected anemia and maintained stable Hb levels within the recommended target range in non-dialysis CKD patients compared with DA (0.45 μg/kg once weekly or 0.75 μg/kg once every two weeks) [11]. Although the half-life of DA is shorter than that of CERA, Roger *et al.* indicated that DA once monthly (1.5 μg/kg) was non-inferior to administration once every two weeks (0.75 μg/kg) for anemia correction in patients with CKD who were not on dialysis [12].

It has been reported that conversion from subcutaneous DA once weekly to subcutaneous CERA once monthly in predialysis CKD patients can efficaciously maintain Hb [13,14]. To improve quality of life, either DA or CERA has been subcutaneously injected once every four weeks to treat anemia in CKD patients who are not on dialysis in Japan. From the perspective of half-life, CERA appears to maintain a stable Hb level, but a controlled trial comparing both CERA and DA once every four weeks has not been reported, to our knowledge. We therefore conducted a randomized controlled trial of both medicines in stable patients who received DA.

## 2. Results and Discussion

In both groups, causes of CKD were glomerulosclerosis (60%) and diabetes mellitus (40%). There were no significant differences in baseline patient characteristics with respect to age, body weight, and the levels of blood pressure, Hb, reticulocytes, white blood cells, platelets, transferrin saturation (TSAT), ferritin, albumin, blood urea nitrogen (BUN), creatinine (Cr), estimated glomerular filtration rate (eGFR), uric acid, and urinary protein excretion rate between both groups (Table 1).

**Figure 1:**
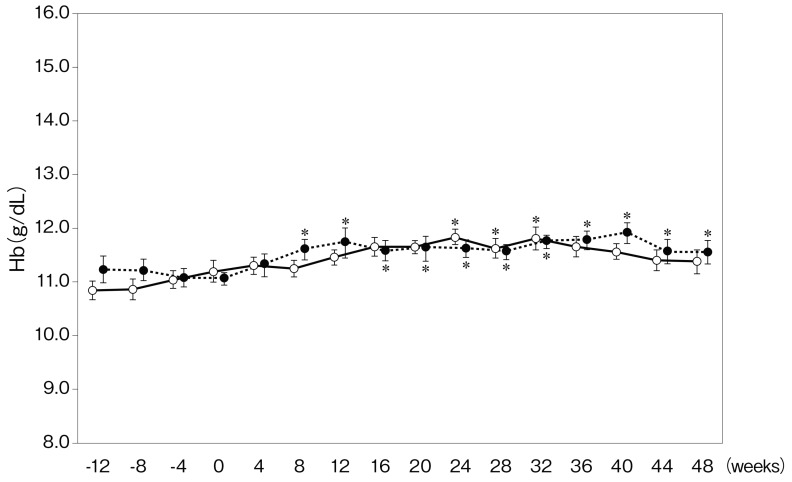
Change in hemoglobin (Hb) concentration. Solid line, darbepoetin α continuation group; dotted line, continuous erythropoietin receptor activator. * *p* < 0.05 *vs.* week 0 in each group. Change in hemoglobin (Hb) concentration. Solid line, darbepoetin α continuation group; dotted line, continuous erythropoietin receptor activator. * *p* < 0.05 *vs.* week 0 in each group. Change in hemoglobin (Hb) concentration. Solid line, darbepoetin α continuation group; dotted line, continuous erythropoietin receptor activator. * *p* < 0.05 *vs.* week 0 in each group.

**Figure 2:**
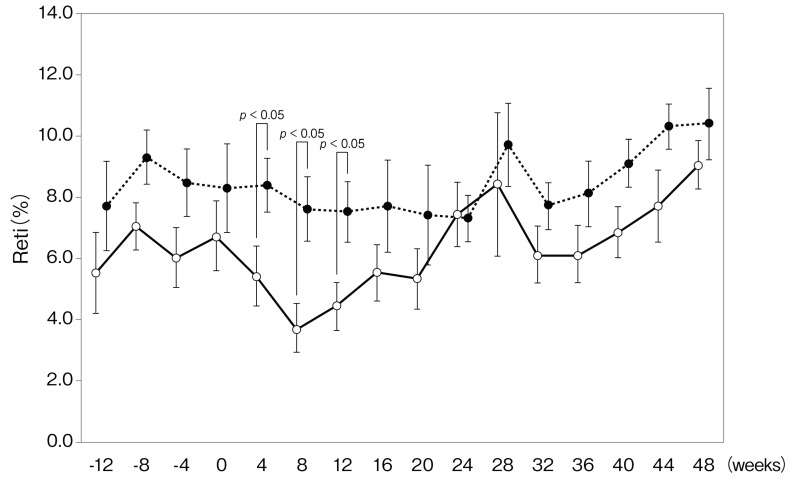
Change in reticulocyte (Reti) concentration. Solid line, darbepoetin α continuation group; dotted line, continuous erythropoietin receptor activator.

In both groups, the rate of achievement of target Hb level (between 11.0 and 12.5 g/dL) in week 0 was 70%. It increased from 70% to 100% in the period between weeks 4 and 48 in both groups, and there was no significant difference between them. Although there was no significant difference in Hb level between both groups, the level of Hb was significantly increased from week 24 compared with week 0 in the DA continuation group, whereas it was increased from week 8 in the CERA changeover group (Figure 1). In addition, the reticulocyte count in the CERA changeover group was significantly increased in week 4 (8.4 ± 0.9 *vs.* 5.4% ± 1.0‰, *p* = 0.043), week 8 (7.6 ± 1.1 *vs.* 3.7% ± 0.8‰, *p* = 0.012), and week 12 (7.5 ± 1.1 *vs.* 4.4% ± 0.8‰ = 0.032) compared with that in the DA continuation group (Figure 2). The doses of DA in the DA continuation group and CERA in the CERA changeover group during the study are shown in Table 2. There was no significant difference in the doses of ESA during the study compared with those given in week 0. Because the total administered doses of DA and CERA over 48 weeks were 0.998 ± 0.106 μg/kg/month (0.250 ± 0.03 μg/kg/week) and 0.956 ± 0.204 μg/kg/month (0.239 ± 0.05 μg/kg/week), respectively, and the Hb levels were similar during the evaluation period, the dose conversion ratio (DCR) was 1.05:1.

Changes to Cr in the range between 0.5 and 1.0 mg/dL or ≥1 mg/dL were seen in 3.3% and 0.8% in the DA continuation group and in 2.5% and 0% in the CERA changeover group, respectively. There was no significant difference in the levels of eGFR between both groups during the study. In addition, there was no significant difference in the levels of blood pressure, white blood cells, platelets, TSAT, ferritin, albumin, BUN, Cr, uric acid, and urinary protein excretion rate between both groups during the evaluation period. Table 3 shows the change of iron status. The number of patients who received oral administration of iron and their total iron doses were same (3 and 11,200 mg) in both groups. The timing of administration was in weeks 12, 20, and 40 in the DA continuation group and weeks 8, 20, and 40 in the CERA changeover group. There was no hospitalization in both groups.

**Table 1:** Patient baseline characteristics.

Patient Characteristic	DA Continuation Group (*n* = 10)	CERA Changeover Group (*n* = 10)	*p* Value
Age (years old)	73.4 ± 8.2	69.4 ± 4.0	0.410
Body weight (kg)	51.7 ± 1.9	53.5 ± 3.0	0.623
Systolic blood pressure (mmHg)	134 ± 6	143 ± 8	0.330
Diastolic blood pressure (mmHg)	72 ± 5	73 ± 4	0.864
Hemoglobin level (g/dL)	11.2 ± 0.2	11.1 ± 0.1	0.615
Reticulocytes (%)	6.7 ± 1.2	8.3 ± 1.5	0.421
White blood cells (/μL)	5190 ± 397	6160 ± 709	0.253
Platelets (×10^4^/μL)	30.0 ± 13.5	30.2 ± 11.8	0.995
Transferrin saturation (%)	33.7 ± 3.0	33.1 ± 3.7	0.910
Ferritin concentration (ng/mL)	171.8 ± 20.8	151.3 ± 16.8	0.452
Albumin concentration (g/dL)	3.9 ± 0.1	3.9 ± 0.1	0.727
Blood urea nitrogen concentration (mg/dL)	51.6 ± 7.2	36.8 ± 4.4	0.101
Creatinine concentration (mg/dL)	3.07 ± 0.38	2.73 ± 0.48	0.585
Estimated glomerular filtration rate (mL/min/1.73 m^2^)	17.3 ± 2.5	21.8 ± 3.4	0.306
Uric acid (mg/dL)	7.0 ± 0.6	5.8 ± 0.4	0.124
Urinary protein excretion rate (g/g Cr)	1.3 ± 0.4	1.5 ± 0.5	0.690

DA: darbepoetin α; CERA: continuous erythropoietin receptor activator.

**Table 2:** Doses of darbepoetin α (DA) and continuous erythropoietin receptor activator (CERA) during the fixed dose period and the evaluation period.

Week	DA Continuation Group	CERA Changeover Group	
	DA Doses (μg)	DA Doses (μg)	CERA Doses (μg)
Week −12	45.0 ± 3.4	41.0 ± 3.8	-
Week −8	45.0 ± 3.4	41.0 ± 3.8	-
Week −4	45.0 ± 3.4	41.0 ± 3.8	-
Week 0	45.0 ± 3.4	-	45.0 ± 3.3
Week 4	49.0 ± 3.7	-	52.5 ± 4.5
Week 8	53.0 ± 4.7	-	50.0 ± 5.3
Week 12	55.0 ± 5.4	-	45.0 ± 10.4
Week 16	53.0 ± 5.6	-	47.5 ± 11.5
Week 20	53.0 ± 5.6	-	47.5 ± 13.7
Week 24	51.0 ± 5.7	-	50.0 ± 13.4
Week 28	53.0 ± 5.6	-	52.5 ± 13.1
Week 32	49.0 ± 8.5	-	52.5 ± 13.1
Week 36	50.0 ± 10.0	-	50.0 ± 13.9
Week 40	53.0 ± 9.4	-	47.5 ± 14.6
Week 44	53.0 ± 9.4	-	52.5 ± 13.1
Week 48	55.0 ± 8.9	-	52.5 ± 13.1

The present study demonstrated that subcutaneous administration of DA and CERA once every four weeks to predialysis patients have a similar effect on achievement of target Hb level (DCR of DA:CERA = 1.05:1) and renal function. However, the number of reticulocytes was significantly increased in the CERA changeover group from week 4 compared with that in the DA continuation group. Additionally, the level of Hb in the CERA changeover group was significantly increased from week 8, which is earlier than that in the DA continuation group (24 weeks), suggesting that longer-acting CERA can increase the Hb level earlier than DA and maintain a constant Hb level.

**Table 3:** Change in iron status during the fixed dose period and the evaluation period.

Week	DA Continuation Group		CERA Changeover Group	
	Transferrin Saturation (%)	Ferritin Concentration (ng/mL)	Transferrin Saturation (%)	Ferritin Concentration (ng/mL)
Week −12	36.3 ± 3.4	172.8 ± 20.6	36.7 ± 4.1	167.7 ± 24.5
Week −8	43.8 ± 7.2	160.2 ± 20.3	32.2 ± 4.5	174.4 ± 29.6
Week −4	35.4 ± 3.9	167.0 ± 19.0	35.3 ± 3.5	164.4 ± 25.7
Week 0	33.7 ± 3.0	171.8 ± 20.8	33.1 ± 3.7	151.3 ± 16.8
Week 4	34.4 ± 3.6	162.2 ± 12.3	36.4 ± 3.2	140.0 ± 16.1
Week 8	33.5 ± 2.5	162.8 ± 16.8	32.5 ± 2.4	146.3 ± 15.7
Week 12	33.2 ± 2.6	148.2 ± 17.1	38.6 ± 4.8	138.6 ± 17.6
Week 16	32.4 ± 2.2	150.9 ± 17.5	33.8 ± 2.5	145.1 ± 16.3
Week 20	33.7 ± 2.8	136.9 ± 15.1	40.4 ± 4.8	172.0 ± 28.8
Week 24	32.7 ± 2.8	161.5 ± 18.6	40.7 ± 3.4	170.3 ± 24.6
Week 28	32.1 ± 3.0	148.8 ± 11.7	39.5 ± 3.7	155.7 ± 20.0
Week 32	36.6 ± 1.7	154.0 ± 10.0	37.6 ± 3.9	160.2 ± 18.5
Week 36	35.2 ± 2.8	156.3 ± 16.3	35.6 ± 2.9	143.9 ± 14.5
Week 40	35.1 ± 2.5	161.2 ± 21.5	29.8 ± 3.2	159.2 ± 25.4
Week 44	28.4 ± 2.6	157.7 ± 19.6	35.2 ± 2.7	161.7 ± 26.0
Week 48	34.0 ± 3.3	163.7 ± 21.2	35.6 ± 3.4	158.6 ± 16.4

DA: Darbepoetin α; CERA: Continuous erythropoietin receptor activator.

Although there are many studies that have compared the effects of DA and CERA on anemia in dialysis patients, only four contain patients not on dialysis. It has been reported that conversion from subcutaneous DA once weekly to subcutaneous CERA once monthly in predialysis CKD patients can efficaciously maintain Hb [13,14]. A switch from DA to CERA maintained Hb levels between 11 and 13 g/dL for 12 months in 37 patients [13] and a switch from epoetin or DA to CERA maintained Hb levels between 10 and 12 g/dL for 24 weeks in 145 patients [14]. In addition, a switch from DA (frequency: 0.81 per month) to CERA (frequency: 0.72 per month) maintained Hb levels between 11 and 12 g/dL for 6 months in 82 patients [15]. The ARCTOS study demonstrated that anemia can be corrected in ESA-naïve patients who have CKD at the predialytic stage with either subcutaneous CERA (starting dosage 0.6 μg/kg every two weeks) or subcutaneous DA (starting dosage 0.45 μg/kg per week). Median dosage in the CERA and DA groups decreased during the course of the study, reaching 0.34 μg/kg every two weeks for CERA and 0.19 μg/kg per week for DA by the end of the evaluation period [10]. In the present study, the doses of CERA and DA were 0.478 μg/kg every two weeks and 0.250 μg/kg per week, respectively.

With respect to the dosage of CERA when switching from DA in non-dialysis patients, the initial conversion dosage when switching from subcutaneous DA to subcutaneous CERA was 75 μg/kg/month of CERA if DA was below 20 μg/kg/week, 100 μg/kg/month of CERA if DA was 21–40 μg/kg/week [13], and 120 μg/kg/month CERA if DA was above 40 μg/kg/week [14]. It has been recently reported that the DCR in switching from DA to CERA in non-dialysis patients is 1.08:1. However, a few problems existed in that study. The stability of Hb level and dosage of DA before study initiation were unknown, and injection frequency was significantly different (DA 0.81 ± 0.30 per month *vs.* CERA 0.72 ± 0.25 per month, *p* < 0.003). In addition, it was an observational study [15]. The present study was an interventional prospective study with stable Hb levels and fixed dosage of DA in the 12 weeks before study initiation, and the DCR between DA and CERA (given subcutaneously once every four weeks) was 1.05:1. Because the range of DA doses in the four weeks before study was 20 to 60 μg, there is a possibility that a higher DCR is needed for patients who were administered more than 120 μg.

DA has been generally administered once every week or once every two weeks for correction of anemia and once every two weeks or once every month for the maintenance of Hb levels in CKD patients who are not on dialysis. On the other hand, CERA has been generally administered once every two weeks or once every month for correction of anemia and maintenance of Hb levels in CKD patients who are not on dialysis because of the longer half-life. A double-blind, randomized, active-controlled study demonstrated that DA once every month was non-inferior to DA once every two weeks for correction of anemia in CKD patients who were not on dialysis [12]. The administration of ESA once every four weeks results in an improvement of quality of life. From the perspective of half-life, CERA appears to maintain stable Hb levels, but a controlled trial that gave both DA and CERA once every four weeks has not been reported. Iron management is essential because iron deficiency can seriously blunt erythropoiesis and impair the effectiveness of ESA. The present study indicated that subcutaneous administration of DA or CERA once every four weeks, along with appropriate administration of an iron supplement, to predialysis patients have similar effects on achievement of target Hb levels, although longer-acting CERA can increase the Hb level earlier than DA and maintain Hb levels. In addition, the reticulocyte counts at weeks 4, 8, and 12 in the CERA changeover group were significantly increased compared with those in the DA continuation group. These significant changes were not related to the ESA dose and iron status. However, it is possible that these superior effects of CERA were due to incorrect DCR.

Randomized, prospective, controlled trials [5,6,7] demonstrated the safety and efficacy of ESA in the correction of anemia in predialysis patients without adverse effects on renal function. We have rarely encountered patients whose renal dysfunction progressed after the administration of ESA. Among the patients in the present study, a Cr increase of 0.5–1.0 mg/dL occurred seven times, and a Cr increase of ≥1 mg/dL occurred once. Further progression of renal dysfunction did not occur in any patient with the control of ESA dosage based on two consecutive Hb and Cr assessments every four weeks.

The limitations of this study were involvement of a single center and the small number of participants. Few patients were available for selection because most patients prefer to visit once per calendar month (once every four or five weeks depending on the month) rather than regularly once every four weeks.

In summary, subcutaneous administration of DA or CERA once every four weeks to predialysis patients has similar effects on achievement of target Hb levels without causing a deterioration of renal dysfunction.

## 3. Experimental Section

Twenty patients over the age of 20 with CKD at the pre-dialysis stage who understood the purpose of the research and gave consent after receiving an explanation, and who had fulfilled specific requirements, were selected. Those requirements were prior subcutaneous administration of a fixed dose of DA for over 24 weeks at a frequency of once every four weeks until registration, and Hb levels >9.5 g/dL and ferritin levels >80 ng/mL in the 12 weeks prior to the study. Patients who had received a blood transfusion within the previous 24 weeks were excluded. The study protocol adhered to the tenets of the Declaration of Helsinki and was approved by the ethics committee of the Nihon University School of Medicine (RK-120608-7, 9 July 2013). This study is registered in the University Hospital Medical Information Network (number 000008531). All patients provided written, informed consent.

### 3.1. Study Design

**Figure 3:**
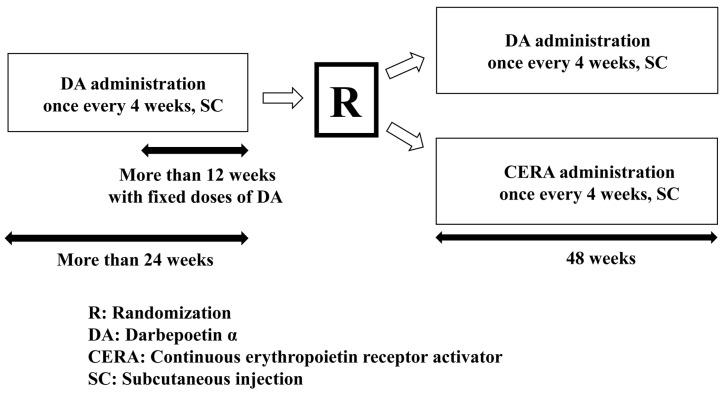
Study protocol.

The present study was an open-label, randomized, single-center, DA-controlled, parallel-group study to determine whether subcutaneous CERA, administered once every four weeks, was more effective for anemia correction than subcutaneous DA once every four weeks in patients who had CKD and were not on dialysis. The duration of the study was 48 weeks (Figure 3). The initial dosages for patients who were randomly assigned to subcutaneous DA or CERA are shown in Table 4. The dose of DA and the corresponding number of patients, respectively, in the four weeks before the study in the DA continuation group were 20 μg and 0 patients, 30 μg and 1 patient, 40 μg and 6 patients, and 60 μg and 3 patients, and in the CERA changeover group were 20 μg and 1 patient, 30 μg and 1 patient, 40 μg and 6 patients, and 60 μg and 2 patients. There was no significant difference between the groups (45.0 ± 3.4 μg *vs.* 41.0 ± 3.8 μg, *p* = 0.443).

**Table 4:** Initial dose of erythropoiesis-stimulating agent.

Dose of DA in the Four Weeks before the Study (μg)	Initial Dose (μg)	
	DA Continuation Group	CERA Changeover Group
20	20	25
30	30	25
40	40	50
60	60	50

DA: darbepoetin α. CERA: continuous erythropoietin receptor activator.

These are based on a report stating that DCR in the subcutaneous administration of DA and CERA once every month to predialysis patients was 1.08:1 [15], and 20, 30, 40, 60, 120, and 180 μg of DA preparations and 25, 50, 75, 100, 150, and 250 μg of CERA preparations are available in our institute. The target range of Hb level was from 11.0 to 12.5 g/dL, and dosing adjustment principles for DA and CERA are shown in Table 5.

The need for dosage adjustment was based on two consecutive Hb and creatinine (Cr) assessments. Dose increase or decrease was set at increments of 20 μg for the DA continuation group and 25 μg for the CERA changeover group. Iron supplementation during the evaluation period was initiated or intensified in case of iron deficiency (serum ferritin < 100 ng/mL) and was temporarily discontinued in patients with serum ferritin >200 ng/mL until serum ferritin returned to below 100 ng/mL. Iron supplementation was administered orally (sodium ferrous citrate 100 mg per day). The study consisted of a 24-week correction period followed by a 12-week period where the dose of DA was fixed and the Hb level was stable, and a 48-week evaluation period. Body weight, blood pressure, and levels of Hb, reticulocytes, white blood cells, platelets, blood urea nitrogen (BUN), serum Cr, uric acid, iron, unbound iron binding capacity, ferritin, albumin, and urinary protein excretion rate and urinary Cr were measured. Estimated glomerular filtration rate (eGFR) and transferrin saturation (TSAT) were calculated every four weeks.

Primary endpoints were rate of achievement of target Hb and change of eGFR. Secondary endpoint was change in Hb level after the start of the study.

**Table 5:** Dosing adjustment principles.

Hemoglobin Level	Increase of Hemoglobin Level in Four Weeks	Increase of Hemoglobin Level in Four Weeks	Dosing Adjustment of Erythropoiesis-Stimulating Agent *
13 g/dL or more	-	-	Interruption of treatment **
12.5 g/dL or more up to 13.0 g/dL	-	-	Dose reduction
Over 11.0 g/dL but under 12.5 g/dL	Under 1.0 g/dL	Under 0.5 mg/dL	Same dose
		Over 0.5 mg/dL but under 1.0 mg/dL	Same dose
		Over 1.0 mg/dL	Lower dose
	Over 1.0 g/dL	Under 0.5 mg/dL	Same dose
		Over 0.5 mg/dL but under 1.0 mg/dL	Lower dose
		Under 0.5 mg/dL	Lower dose
Under 11.0 g/dL	Under 1.0 g/dL	Under 0.5 mg/dL	Higher dose
		Over 0.5 mg/dL but under 1.0 mg/dL	Higher dose
		Over 1.0 mg/dL	Same dose
	Over 1.0 g/dL	Under 0.5 mg/dL	Higher dose
		Over 0.5 mg/dL but under 1.0 mg/dL	Same dose
		Under 0.5 mg/dL	Same dose

* Dose increase/decrease was set at increments of 20 μg for the darbepoetin α continuation group and 25 μg for the continuous erythropoietin receptor activator changeover group; ** Administration is resumed at one dose increment lower when hemoglobin concentration falls below 12.5 g/dL.

### 3.2. Statistical Analyses

Results are expressed as mean ± standard error (SE) values. We assessed differences in the same group using the *t*-test for paired data. The unpaired *t*-test was used to compare mean values between groups. Repeated ANOVA was used to compare median changes between groups. SPSS Statistics ver. 20 (IBM Japan, Tokyo, Japan) was used for statistical analysis. Significance was established at a level of *p* < 0.05.
